# Papillary thyroid cancer with extrathyroidal extension of desmoid-type fibromatosis. A case report of an aggressive presentation of an uncommon pathologic entity

**DOI:** 10.1016/j.ijscr.2019.08.001

**Published:** 2019-08-08

**Authors:** Eve M. Roth, Courtney E. Barrows, Michiya Nishino, Barry Sacks, Per-Olof Hasselgren, Benjamin C. James

**Affiliations:** a Department of Surgery, Beth Israel Deaconess Medical Center, Harvard Medical School, Boston, MA, United States; b Department of Pathology, Beth Israel Deaconess Medical Center, Harvard Medical School, Boston, MA, United States; c Department of Radiology, Beth Israel Deaconess Medical Center, Harvard Medical School, Boston, MA, United States

**Keywords:** FNA, fine needle aspiration, PTC, papillary thyroid cancer, PTC-DTF, papillary thyroid cancer with desmoid-type fibromatosis, Papillary thyroid cancer with desmoid-type fibromatosis (PTC-DTF), Local infiltration, Recurrence, Management

## Abstract

**Introduction:**

Papillary thyroid cancer with desmoid-type fibromatosis (PTC-DTF) is an uncommon tumor characterized by extensive stromal proliferation of fibroblasts and myofibroblasts with a small component of PTC. We report a case of PTC-DTF with infiltration of the mesenchymal component of tumor into perithyroidal muscle and early recurrence of desmoid after thyroidectomy, an outcome previously not reported.

**Presentation of case:**

A 20-year-old man underwent left hemithyroidectomy for a thyroid nodule. Pathology demonstrated a 4.2 cm tumor with PTC-DTF with the PTC comprising <10% of the tumor. The stromal component extended into adjacent skeletal muscle. After completion thyroidectomy, histopathology of the right thyroid lobe revealed no malignancy or fibromatosis. Neck MRI 16 months after the initial operation revealed a 10.5 cm tumor in the left thyroid bed. Core biopsy and open excisional biopsy showed desmoid-type fibromatosis without PTC. The patient is undergoing chemotherapy of his recurrent desmoid-type fibromatosis.

**Discussion:**

In patients with PTC-DTF there is a risk of recurrence of the benign component of the tumor. In recent reports, the role of less aggressive surgery, or even non-surgical management, of patients with recurrent DTF has been emphasized, in particular when extensive surgery may be associated with high risk of functional loss. The management of our patient adheres to modern recommendations for the treatment of DTF.

**Conclusion:**

Patients with PTC-DTF should be carefully monitored after thyroidectomy for both recurrent PTC and local recurrence of the fibrous component of the tumor.

## Highlights

• Papillary thyroid cancer with desmoid-type fibromatosis (PTC-DTF) is an uncommon variant of PTC.
• The stromal component of the tumor may have an aggressive behavior with risk for local recurrence.
• Management of recurrent desmoid-type fibromatosis on the neck can be challenging.
• Patients with PTC-DTF need to be monitored carefully after thyroidectomy for local recurrence of the stromal component of the tumor.

## 1 Introduction

Papillary thyroid cancer (PTC) with fibromatosis- or nodular fasciitis-like stroma is an uncommon variant of PTC initially described almost three decades ago [[1], [2], [3], [4]]. The tumor is characterized by extensive stromal proliferation of fibroblasts and myofibroblasts with a small (typically <20%) component of PTC. Although PTC with fibromatosis-like stroma may be associated with more aggressive clinical features than PTC with nodular fasciitis-like stroma, the WHO Classification of Tumors considers the two subtypes of PTC identical and the two names are often used interchangeably [5].

Aberrant nuclear expression of β-catenin in the mesenchymal cells is present in most cases of PTC with fibromatosis-like stroma [6,7]. Because a similar pattern of β-catenin expression is seen in desmoid-type fibromatosis in other tissues [[8], [9], [10], [11]], it was recently proposed that PTC with fibromatosis- or nodular fasciitis-like stroma should be renamed PTC with desmoid-type fibromatosis (PTC-DTF) [6,7].

We report a case of PTC-DTF with infiltration of desmoid-type fibromatosis into perithyroidal muscle and an early recurrence of the mesenchymal tumor component after thyroidectomy, an outcome previously not reported in the literature. The case report adheres to the Surgical CAse REport (SCARE) guidelines [12].

## 2 Case report

**Fig. 1:**
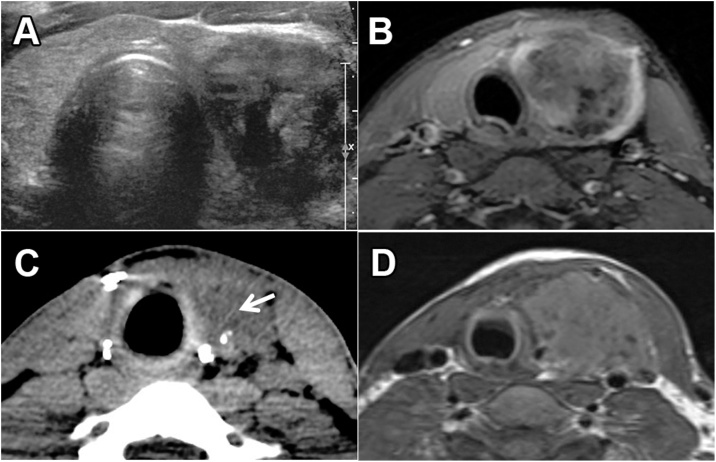
(A) Neck ultrasound revealing a 3.2 × 2.7 × 1.9 cm heterogenous nodule in the left thyroid lobe. (B) Neck MRI performed before left hemithyroidectomy showing no evidence of invasion of the left thyroid tumor into surrounding tissues. (C) Neck CT performed 3 months after completion thyroidectomy revealing a 2 cm fluid accumulation in the left thyroid bed (arrow) but no evidence of tumor recurrence. (D) Neck MRI revealing a 10.5 cm tumor in the left thyroid bed 16 months after the initial left hemithyroidectomy.

A 20-year-old man without personal or family medical history presented to his primary care physician with a left-sided thyroid nodule. Ultrasound showed a 3.2 × 1.9 × 2.7 cm heterogeneous nodule in the left thyroid lobe without calcifications or increased vascularity (Fig. 1A). Fine needle aspiration (FNA) yielded a mixture of spindle cells and epithelioid histiocytes classified as benign and suggestive of granulomatous inflammation. A 6-month follow-up ultrasound revealed growth of the nodule to 3.9 cm in its largest dimension, prompting surgical referral. Physical examination at that time revealed a 4-cm firm mass in the left thyroid lobe with mild left-sided neck induration. The patient had no palpable lymphadenopathy or right-sided thyroid nodules. He was clinically and biochemically euthyroid. The firmness of the mass and the neck induration raised concern for aggressive thyroid cancer. Repeat FNA yielded a hypocellular specimen with mildly atypical spindle and epithelioid cells classified as “atypical – a spindle cell neoplasm cannot be excluded.” The patient was recommended a diagnostic left hemithyroidectomy. Because of the worrisome physical examination, preoperative neck MRI was performed, confirming left-sided thyroid nodule but no obliterated tissue planes or invasion into adjacent structures (Fig. 1B). The right thyroid lobe appeared normal. During surgery, a firm 4–5 cm mass replacing most of the left thyroid lobe was encountered with surrounding inflammatory changes. There was no central compartment lymphadenopathy and no residual gross disease was present at the end of the hemithyroidectomy. Pathology demonstrated a 4.2 cm tumor with desmoid-type fibromatosis and PTC comprising <10% of the tumor (described in detail below). The stromal component extended outside the thyroid into adjacent skeletal muscle. Surgical margin was positive for the fibromatosis-like component.

The patient subsequently underwent completion thyroidectomy. Histopathology of the right thyroid lobe revealed no malignancy or fibromatosis. The patient had an uneventful recovery after completion thyroidectomy and was doing well 3 and 6 months postoperatively with well-healed neck incision. However, the ventral neck was firmer than typically seen after a thyroidectomy. Because of this, a neck CT was performed 3 months after completion thyroidectomy, revealing a 2 cm fluid accumulation in the left surgical bed as well as obscuration of adjacent fat planes, consistent with postoperative changes (Fig. 1C). There was no evidence of recurrent tumor.

Because of risk for local recurrence of the desmoid-type fibromatosis, the patient was scheduled for neck MRI but was lost for follow up. He returned 16 months after the initial operation with a palpable mass in his left neck and an MRI revealing a 10.5 cm tumor in the left thyroid bed (Fig. 1D). Core biopsy and open excisional biopsy revealed desmoid-type fibromatosis without recurrent PTC. After multidisciplinary evaluation, the patient is undergoing treatment with doxorubicin for his recurrent desmoid-type fibromatosis in line with recent recommendations in the literature [[8], [9], [10], [11]]. After two cycles of doxorubicin treatment, a neck MRI showed that no further significant growth of the tumor had occurred. The trachea remained deviated to the right but there was no tracheal compression. The patient had no airway symptoms and a spirometry was within normal limits.

## 3 Left thyroid lobe pathology

**Fig. 2:**
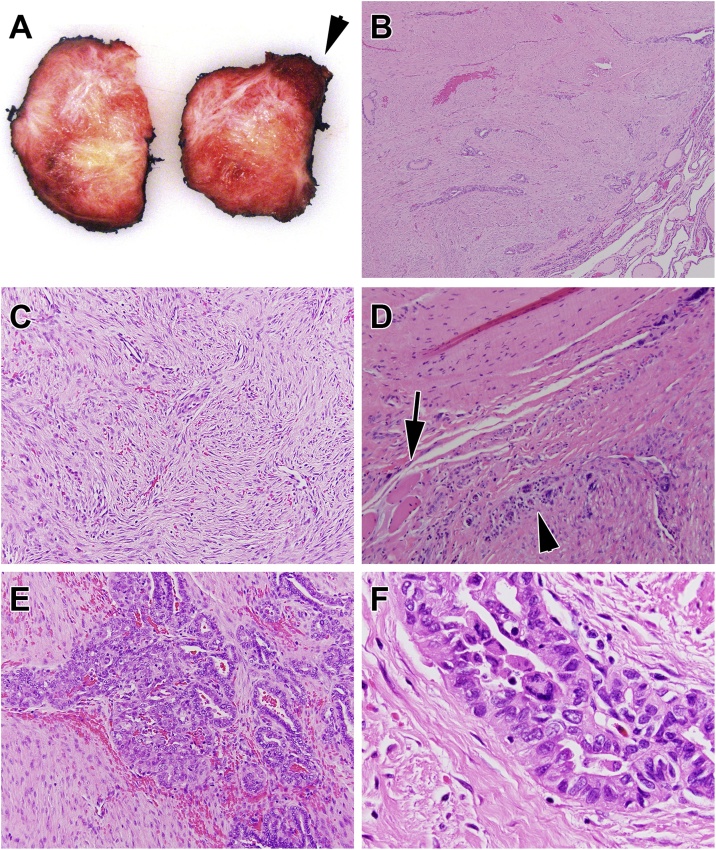
Macroscopic and microscopic findings from left thyroid lobectomy. (A) Cut sections of thyroid demonstrating a whorled fibrotic nodule. The arrowhead shows thyroid parenchyma uninvolved by tumor. (B) Low-magnification (40×) image of interface between the tumor and adjacent thyroid parenchyma (lower right corner of panel). (C) Stromal component comprising over 90% of the tumor (100× magnification). (D) Extrathyroidal portion of tumor (lower right corner of panel) infiltrating skeletal muscle (arrow). Atrophic skeletal muscle fibers are present (arrowhead) (100× magnification). (E) Epithelial component (PTC) of the tumor and its relationship to the stromal component (100× magnification). (F) High-magnification (400×) of the PTC component of the tumor. Note the nuclear enlargement, nuclear irregularity, and chromatin pallor. Panels B–F stained with hematoxylin & eosin.

On gross examination, cut section of the left thyroid lobe showed a circumscribed yellow to white 4.2 × 3.9 × 3.1 cm fibrotic nodule with areas of focal hemorrhage (Fig. 2A). Microscopically, the tumor had two components. The stromal component comprised >90% of the lesion and consisted of fibroblastic/myofibroblastic proliferation, consistent with desmoid-type fibromatosis (Fig. 2B and C). The stromal component extended outside the thyroid with microscopic infiltration into adjacent skeletal muscle (Fig. 2D), and was present at the inked surgical margin. The PTC comprised <10% of the tumor and was confined to the thyroid with negative surgical margins (Fig. 2E). The epithelial component demonstrated characteristic cytological features of PTC, including nuclear enlargement, elongation, grooves, irregular contours, and chromatin pallor (Fig. 2F). No metastatic lymph nodes were identified.

**Fig. 3:**
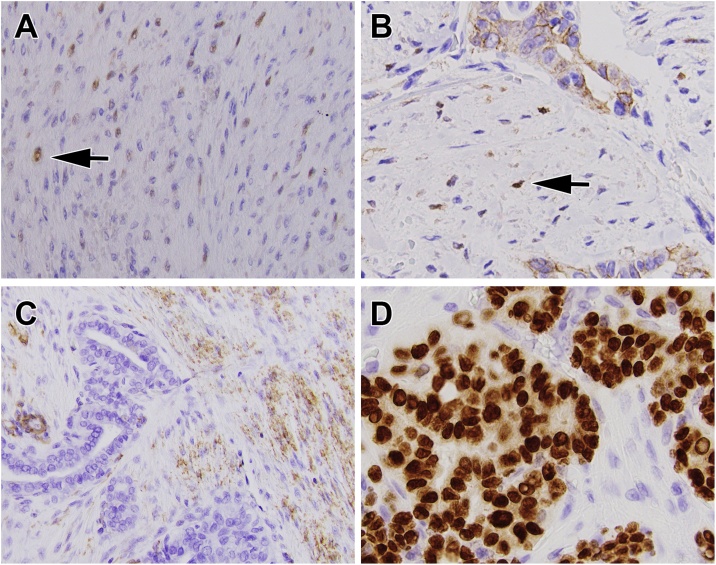
Immunohistochemical staining from the left thyroid tumor. (A) Beta-catenin immunostain, showing aberrant nuclear staining (arrow) in many of the stromal cells (200× magnification). (B) Beta-catenin immunostain, showing aberrant nuclear staining in the stromal cells (arrow). The epithelial (papillary carcinoma) component of the tumor shows membranous staining for beta-catenin without aberrant nuclear localization of the protein (400× magnification). (C). Smooth muscle actin immunostain highlights the stromal component of the tumor (200× magnification). (D) TTF-1 immunostain highlights the PTC nuclei (400× magnification).

Immunohistochemistry showed aberrant nuclear staining for β-catenin in stromal cells whereas the epithelial component showed membranous staining for β-catenin without aberrant nuclear expression (Fig. 3A and B). Stromal cells were positive for smooth muscle actin (Fig. 3C) and desmin (not shown). In the PTC, the nuclei were positive for TTF-1 (Fig. 3D), PAX-8, and cytokeratin (not shown). The PTC was negative for calcitonin, smooth muscle actin, desmin, S100, estrogen and androgen receptors. The stromal component was negative for cytokeratin (AE1/AE3, Cam 5.2), S100, TTF-1, PAX-8, estrogen and androgen receptors. A monoclonal antibody (VE1) for the BRAF V600E mutation was negative in the epithelial and stromal components of the tumor.

## 4 Discussion

PTC-DTF may present as a firm enlarging thyroid mass [6,7]. Because the PTC component of these tumors is typically <10–20%, cytology after FNA may be false-negative for malignancy. In previous reports, cytology ranged from benign [13] to concerning for spindle-cell neoplasms [14,15] or diffuse sclerosing PTC [16,17]. In a recent report of 14 patients with PTC-DTF, cytology was suspicious or positive for cancer in the majority of cases [7]. In our patient, the initial cytology was consistent with a benign nodule and repeat cytology was described as “atypical - a spindle cell lesion cannot be excluded.”

Aberrant nuclear expression of β-catenin in the spindle cells was observed in several previous cases of PTC-DTF [6,7,18] reported by Na et al. [18], similar to DTF in other tissues [[8], [9], [10], [11]]. An activating mutation in the β-catenin-encoding *CTNNB1* gene, as reported in the stromal cells of PTC-DTF [6], suggests altered Wnt/β-catenin-dependent pathway in these tumors.

DTF may be associated with mutations of the adenomatous polyposis coli (APC) gene and familial adenomatous polyposis (FAP) [9,10]. Our patient did not have a personal or family history of FAP but the patient and family members have not been tested for APC gene mutations.

The pathogenesis of stromal proliferation in PTC-DTF is incompletely understood. While reactive and neoplastic etiologies have been proposed [[1], [2], [3], [4]], changes in the Wnt/APC/β-catenin pathway suggest a primary genetic alteration rather than a reactive process.

Despite the small size of PTC in PTC-DTF, cases with extrathyroidal extension of the PTC component and lymph node metastases have been described [2,7]. Interestingly, lymph nodes containing metastases from PTC harbored DTF in about 30% [7]. In our patient, extrathyroidal extension of PTC and lymph node metastases were absent and the contralateral lobe was without malignancy. Therefore, the prognosis with regards to the PTC should be favorable.

In contrast, the mesenchymal component of the tumor presented challenges regarding management and risk for recurrence. Extrathyroidal extension of DTF with infiltration into surrounding skeletal muscle has been reported previously [2,6] but local recurrence has not been described before. Although neck CT seven months after the initial operation did not show recurrence in our patient, MRI nine months later revealed a large mass in the left thyroid bed, ultimately proven to be DTF.

Because of the rarity of PTC-DTF, guidance for initial management and treatment of recurrence must be sought in the literature on primary DTF in other tissues. Although earlier reports emphasized the importance of negative surgical margins to prevent local recurrence [19], recent studies suggest that a positive margin does not always correlate with recurrence [9,11,20]. These observations have led to recommendations to avoid aggressive surgery to achieve negative margins, particularly if surgery could result in unnecessary functional loss [[8], [9], [10], [11]]. Additionally, surgical trauma may in itself stimulate growth of DTF [8]. In our patient, the decisions to avoid reoperation for positive margin and aggressive surgery for local recurrence reflected these recommendations [[8], [9], [10], [11]].

The shift towards conservative surgical, and even non-surgical, management of DTF is supported by the fact that some of these tumors are “self-limiting” and may regress spontaneously [[8], [9], [10], [11]]. Many DTF tumors respond to nonsteroidal anti-inflammatory drugs, hormone treatment, chemotherapy, or in resistant cases, radiation therapy [[8], [9], [10], [11]]. Our patient is presently undergoing chemotherapy by a team of oncologists specializing in the care of mesenchymal tumors, including desmoids.

## 5 Conclusion

Although the patient is still undergoing treatment for his recurrent DTF, precluding statement about the long-term outcome, the present report is important for raising awareness of an extremely rare condition and highlighting recent management options for PTC-DTF that shows aggressive features with local recurrence of the mesenchymal tumor component. Patients with this unusual tumor should be carefully monitored after thyroidectomy for both recurrent PTC and local recurrence of the fibrous component of the tumor. In patients with recurrent DTF, a multidisciplinary approach is essential.

## Funding

This research did not receive any specific grant from funding agencies in the public, commercial, or not-for-profit sectors.

## Ethical approval

The study has been reviewed by the Committee on Clinical Investigations (CCI) at the Beth Israel Deaconess Medical Center, Harvard Medical School, Boston, MA, USA. The IRB protocol is 2019D000498. “The proposed activity as described DOES NOT constitute human subjects research. Therefore, no further CCI review and approval is required.” The date of Review and Determination was 6/3/2019.

## Consent

The individual described in the case report cannot be identified by any of the images or through any part of the text.

Written informed consent was obtained from the patient for publication of this case report and accompanying images. A copy of the written consent is available for review by the Editor-in-Chief of this journal on request.

## Author contribution

Study concept and design: Eve Roth, Courtney Barrows, Per-Olof Hasselgren, Benjamin James.

Data collection: Eve Roth, Michiya Nishino, Barry Sacks.

Interpretation of data: Eve Roth, Courtney Barrows, Michiya Nishino, Barry Sacks, Per-Olof Hasselgren, Benjamin James.

Initial drafting of paper: Eve Roth, Per-Olof Hasselgren, Benjamin James.

## Registration of research studies

Research Registry UIN 4950.

## Guarantor

Per-Olof Hasselgren.

Benjamin James.

## Provenance and peer review

Not commissioned, externally peer-reviewed

## Declaration of Competing Interest

None of the authors have any conflict of interest to declare.
